# MERS-CoV Antibodies in Humans, Africa, 2013–2014

**DOI:** 10.3201/eid2206.160064

**Published:** 2016-06

**Authors:** Anne Liljander, Benjamin Meyer, Joerg Jores, Marcel A. Müller, Erik Lattwein, Ian Njeru, Bernard Bett, Christian Drosten, Victor Max Corman

**Affiliations:** International Livestock Research Institute, Nairobi, Kenya (A. Liljander, J. Jores, B. Bett);; University of Bonn Medical Centre, Bonn, Germany (B. Meyer, M.A. Müller, C. Drosten, V.M. Corman);; University of Bern, Vetsuisse Faculty, Bern, Switzerland (J. Jores);; EUROIMMUN AG, Lübeck, Germany (E. Lattwein);; Ministry of Health, Nairobi, Kenya (I. Njeru);; German Centre for Infection Research, Bonn (C. Drosten, V.M. Corman)

**Keywords:** Middle East respiratory syndrome, coronavirus, MERS-CoV, antibodies, livestock handlers, humans, eastern Kenya, Africa, viruses

## Abstract

Dromedaries in Africa and elsewhere carry the Middle East respiratory syndrome coronavirus (MERS-CoV). To search for evidence of autochthonous MERS-CoV infection in humans, we tested archived serum from livestock handlers in Kenya for MERS-CoV antibodies. Serologic evidence of infection was confirmed for 2 persons sampled in 2013 and 2014.

Middle East respiratory syndrome coronavirus (MERS-CoV) infection causes severe respiratory illness in humans. Some cases have been sporadic, but others have been part of nosocomial outbreaks mainly on the Arabian Peninsula (*1*), where dromedary camels widely carry the virus and human infections have been directly linked to contact with camels (*2*–*4*). As of January 2016, at least 1,625 acute cases in humans and 586 deaths from MERS-CoV infection have been documented (*5*). In a geographically comprehensive, age-stratified sample representing the population of Saudi Arabia, antibodies against MERS-CoV were detected in ≈0.15%, indicating sporadic infections without severe disease (*6*).

Antibodies against MERS-CoV have also been detected in dromedaries in several countries in Africa (e.g., Nigeria, Egypt, Kenya) in samples collected as long as 30 years ago (*7*–*10*). East Africa harbors >70% of the world’s dromedary population; predominantly unilateral trade is conducted from Africa to the Arabian Peninsula (*11*). The basal phylogenetic clustering of viral sequences from camels in Africa suggests an African origin of MERS-CoV (*9*,*10*). 

To our knowledge, evidence for autochthonous human infections in Africa has not been reported. To search for evidence of previous MERS-CoV infection, we tested archived human serum samples collected from 1,122 livestock handlers in Kenya during 2013 and 2014. This work was done in compliance with national regulations and was approved by the ethical committee of African Medical Research and Foundation, Kenya (AMREF-ESRC P65/2013).

## The Study

The serum samples were collected as part of a household survey conducted during 2013–2014 in 2 eastern counties of Kenya, Garissa and Tana River (Technical Appendix). Of those for whom information about sex and age was known, 603 were female and 407 were male, and median age was 27 (range 5–90) years. Occupational data were available for 650 (57.9%) participants; the 3 largest occupational groups represented were pastoralist (20.6%), farmer (17.0%), and student (11.4%). The households of nearly all participants kept or owned livestock, mainly goats, sheep, cattle, and donkeys. Although camel husbandry was not common among participants, camels are widespread in this region. The average camel density (calculated on the basis of census data from 2000–2013) was 1.68 and 1.98 camels/km^2^ in Garissa and Tana River County, respectively (*7*).

**Figure 1:**
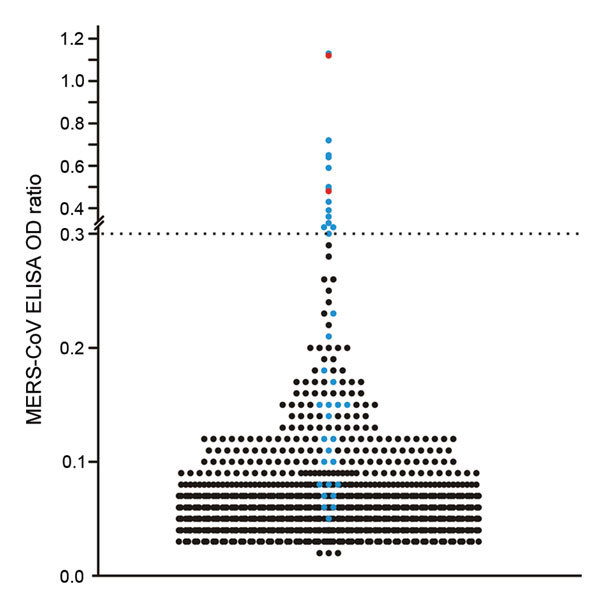
Plot of all individual optical density (OD) ratios obtained from recombinant ELISA testing of human serum samples for Middle East respiratory syndrome coronavirus (MERS-CoV) antibodies, Africa, 2013–2014. All 16 samples exceeding the cutoff of 0.3 and 22 other samples showing an OD ratio below the cutoff were subsequently tested in a plaque-reduction virus neutralization (PRNT) test; these samples are shown in blue, and the 2 samples positive by PRNT are shown in red. The horizontal dashed line represents the cutoff value as determined in a nationwide, cross-sectional serologic study in Saudi Arabia (*6*).

We analyzed serum samples for antibodies against MERS-CoV by using a commercial anti–MERS-CoV recombinant ELISA (rELISA; EUROIMMUN AG, Lübeck, Germany), which is based on the recombinant MERS-CoV spike protein subunit 1 and specifically detects IgG. Samples were tested at a dilution of 1:100; an optical density ratio of 0.3 was set as a cutoff (*6*,*12*). The assay conditions used were the same as those used during a nationwide serologic study in Saudi Arabia (*6*). A total of 16 (1.40%) samples had positive results by rELISA (Table, Figure 1). The proportion of seropositive specimens in both counties in Kenya did not differ significantly (Fisher exact test, p = 0.07).

**Table:** Seropositivity for Middle East respiratory syndrome coronavirus in samples from humans in Kenya, 2013–2014*

County	No. samples tested	No. (%) positive by rELISA	No. (%) positive by rELISA and with >50% plaque reduction at 1:20 dilution
Garissa	559	4 (0.72)	0
Tana River	563	12 (2.13)	2 (0.36)
Total	1,122	16 (1.43)	2 (0.18)

*rELISA, recombinant ELISA.

We subsequently tested all samples positive by rELISA by using a highly specific MERS-CoV plaque-reduction neutralization test (PRNT) as recommend by the World Health Organization (*6*,*13*). Of note, the MERS-CoV strain EMC/2012 used for PRNT may genotypically differ from putatively circulating MERS-CoV strains from Africa. However, there is no serotypic discrimination between strains because the ability of human serum to neutralize diverse MERS-CoV strains, including the EMC/2012 strain, does not differ (*14*). 

**Figure 2:**
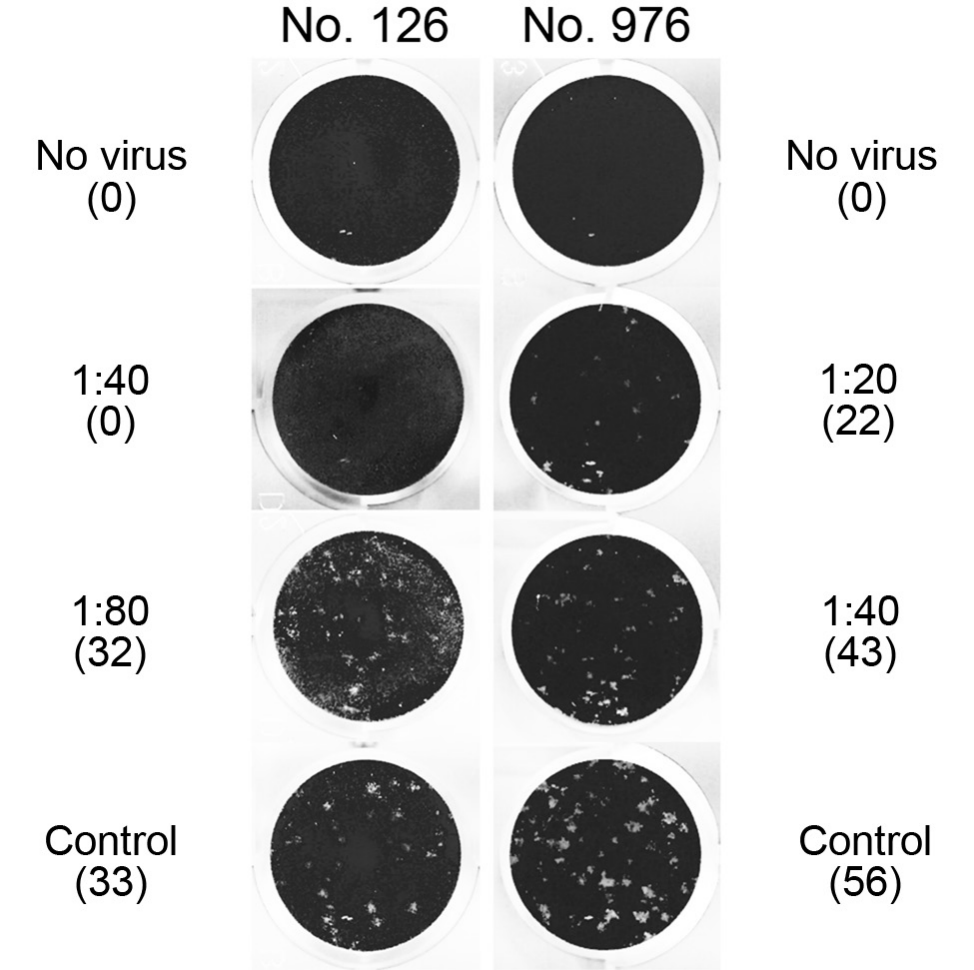
Middle East respiratory syndrome coronavirus (MERS-CoV) plaque-reduction neutralization test (PRNT) results for 2 serum samples positive by recombinant ELISA, showing virus neutralization activity against MERS-CoV strain EMC/2012 exceeding a titer of 1:10. Titers and number of plaques (in parenthesis) are shown next to the corresponding images. Sample no. 976 showed >50% plaque reduction up to a titer of 1:20, and sample no. 126 showed >90% plaque reduction up to a titer of 1:40. No serum was added to the control wells. Note that the image cannot represent the morphology and the contrast of plaques that was visible with direct inspection of cell culture plates with an appropriate light source, as was done for these experiments.

For the PRNT, dilutions starting at 1:10 were used and titers resulting in 50% (PRNT_50_) and 90% (PRNT_90_) plaque reduction were recorded. The 1:20 dilution was the lowest possible diagnostically significant titer (*6*). The PRNT_50_ end point was considered confirmation of positivity by rELISA because this end point was found to be most sensitive and still specific during investigations of antibody responses in reverse transcription PCR–confirmed MERS-CoV–positive samples from patients in South Korea (*14*). Of the 16 samples positive by rELISA, 2 (0.18%) had reproducible MERS-CoV PRNT_50_ titers of 1:20 and 1:40 (Table; Figure 2); 1 of these samples also had a titer of 1:40 when the more stringent PRNT_90_ end-point criterion was used (Figure 2). For controls, we conducted PRNT testing of 22 samples negative by rELISA from persons originating from the same region as the 2 samples positive by PRNT. None of these 22 samples showed neutralizing activity at a 1:20 dilution (Figure 1). 

The 2 samples positive by PRNT were from a woman (26 years of age) and a man (58 years of age) from Tana River County. The woman kept goats, sheep, cattle, and donkeys; the man kept goats and donkeys. Both persons had low antibody titers, and neither reported any recent clinical symptoms, indicating that their MERS-CoV infections probably occurred well before the time of sampling and that the infections may have been mild or subclinical. Because data about persistence of MERS-CoV antibodies after asymptomatic infection are not available, it can only be speculated when and where these infections were acquired. Neither the 2 MERS-CoV antibody–positive persons nor most of the other tested persons owned dromedaries. Nevertheless, camels roam in both counties (*7*), and humans have regular contact with camels and are likely to consume camel products.

Our study has several limitations. First, we were not able to test samples from persons who had close contact with camels, such as camel pastoralists. Second, although we used well-validated methods and a 2-step approach recommended by the World Health Organization for MERS-CoV diagnostics (*13*), our results should be confirmed by larger studies that may enable direct virus detection.

## Conclusions

The absence of autochthonous human MERS-CoV infections in Africa has triggered hypotheses regarding differences in disease transmission between Africa and the Arabian Peninsula and has raised doubts regarding the role of camels as a source of infection. Our study provides evidence for unrecorded human MERS-CoV infections in Kenya. The proportion of seropositive specimens that we found is comparable to previously reported proportions of unrecorded infections in the general population in Saudia Arabia (1.52% vs. 1.43% positivity by rELISA and 0.15% vs. 0.18% positivity by PRNT for Kenya vs. Saudi Arabia, respectively) (*6*). Because of an apparently low infection rate and a bias toward reporting severe cases, the discovery of unreported MERS cases requires testing of large sample sizes with well-validated serologic methods (*6*). Although the number of samples we tested was approximately only one tenth of the number of samples tested during the Saudi Arabia study, the proportion of seropositive specimens may be similar in Kenya and Saudi Arabia. The lack of a well-developed public health system in parts of Africa could lead to underdiagnosis of clinical cases and would therefore prevent case notification. Moreover, less accessible hospital care might preclude large nosocomial outbreaks as have been observed in countries on the Arabian Peninsula and in South Korea. Other possible explanations for the absence of confirmed and reported clinical cases of MERS-CoV infection in Africa include lesser virulence of strains from Africa and cultural differences that might cause persons of different age ranges to be exposed to the virus.

On the basis of the ability of MERS-CoV to infect a wide range of hosts in cell culture experiments (*15*), it remains to be excluded that other wild and livestock animals might act as additional sources of human MERS-CoV infection. It is paramount to characterize MERS-CoVs from humans, camels, or tentative other animal hosts in Africa. For increased understanding of any possible differences in pathogenicity and transmission potential, these MERS-CoV strains should be compared with isolates from the Middle East. 

**Technical Appendix.** Sampling frame and methods used in a household survey conducted during 2013–2014 to compare the risk for zoonotic virus infection among humans and livestock in Eastern Kenya.
