# Supplementary material for: MERS-CoV Antibodies in Humans, Africa, 2013–2014
Source: Emerg Infect Dis. 2016 Jun;22(6):1086–9. doi: 10.3201/eid2206.160064 (PMC4880087; doi:10.3201/eid2206.160064)
Supplement: Supplementary file 1 — Technical Appendix. Sampling frame and methods used in a household survey conducted during 2013–2014 to compare the risk for zoonotic virus infection among humans and livestock in Eastern Kenya. [file 16-0064-Techapp-s1.pdf]

Article DOI: http://dx.doi.org/10.3201/eid2206.160064

# MERS-CoV Antibodies in Humans, Africa, 2013–2014

## Technical Appendix

## Sampling Frame and Method

The study was designed to compare the risk of zoonotic virus infection (e.g., Rift Valley fever virus) in people and livestock in selected pastoral and irrigated areas in Eastern Kenya. The sample size required was determined using the methods described by Dohoo et al. (*1*). This analysis indicated that the study needed to use 110 households and 550 subjects per area, assuming that five subjects would be sampled per household. A sampling framework was prepared by listing all the households in these areas. This was done with the help of the local administrators and the managers of irrigation projects. In total more than 2,500 households were identified. A random sampling technique was subsequently used to select the households, which were included in this study. In each household, the household head and any other person more than 5 years of age were recruited after providing informed consent. In households that had more than 5 members at an age above 5 years, the household head was requested to identify four other persons that could be sampled.

Subjects were informed the study would include screen for Rift Valley fever and other zoonotic viruses. The subjects had been notified that samples would be shipped to external laboratories for specialized screening. Other pathogens than Rift Valley Fever virus to be screened for and causing flu like syndromes (such as MERS-CoV) were not mentioned specifically. Before initiating the MERS-CoV work, an amendment of the already given ethical approval (No: AMREF P65/2013) was given from AMREF's Ethical Review Board to allow for additional screening for MERS-CoV of the bio banked serum samples. Additionally, an approval for the shipment of sample to the lab in Bonn was given by the Kenyan Ministry of Health (No: MOH/ADM/1/1/81/VOL1.1)

After providing an informed consent, a subject was asked to sit in a comfortable position in preparation for sampling. Up to 20 ml venous blood was drawn from subjects above 10 years and 15 ml from those between 5 – 10 years from the median cubital vein after applying a tourniquet 3–4 inches above the injection site and disinfection it with 70% isopropyl alcohol. Sterilized butterfly needles and pre-coded vacutainer tubes were used. Blood samples were collected in Lithium Heparin Tubes (BD Vacutainer®, UK) and in Clot Activator Tubes (BD Vacutainer®, UK) for subsequent analysis. After sampling, punctured sites were bandaged using adhesive tapes. Blood samples collected in Clot Activator Tubes were allowed to clot and later centrifuged at 3,000xg for 10 minutes to harvest serum. The sampling forms filled out at the time of sampling captured the required metadata such as the age, sex, relationship to the household head, the size of the household, animals kept or owned.

All serum samples were transferred into barcoded 2ml cryogenic vials in the field. The samples were kept frozen (using dry ice) until they arrived at the ILRI bio bank in Nairobi where they were transferred to liquid nitrogen tanks for long-term storage. For MERS-CoV screening, samples were thawed at 4°C, aliquoted and shipped to the Institute of Virology in Bonn, Germany at 4°C. After arrival in Bonn, samples were stored at 4°C and immediately subjected to analysis.

**Reference**

1. Dohoo I, Martin W, Stryhn H. Veterinary epidemiologic research, 2nd ed. Charlottetown (Prince Edward Island, Canada): VER Inc.; 2009. p. 33–53.
